# Water Use Practices Limit the Effectiveness of a Temephos-Based *Aedes aegypti* Larval Control Program in Northern Argentina

**DOI:** 10.1371/journal.pntd.0000991

**Published:** 2011-03-22

**Authors:** Fernando M. Garelli, Manuel O. Espinosa, Diego Weinberg, María A. Trinelli, Ricardo E. Gürtler

**Affiliations:** 1 Laboratorio de Eco-Epidemiología, Facultad de Ciencias Exactas y Naturales, Universidad de Buenos Aires, Buenos Aires, Argentina; 2 Fundación Mundo Sano, Buenos Aires, Argentina; 3 Laboratorio de Química del Agua, INQUIMAE and Departamento de Química Inorgánica, Analítica y Química Física, Facultad de Ciencias Exactas y Naturales, Universidad de Buenos Aires, Buenos Aires, Argentina; Mahidol University, Thailand

## Abstract

**Background:**

A five-year citywide control program based on regular application of temephos significantly reduced *Aedes aegypti* larval indices but failed to maintain them below target levels in Clorinda, northern Argentina. Incomplete surveillance coverage and reduced residuality of temephos were held as the main putative causes limiting effectiveness of control actions.

**Methodology:**

The duration of temephos residual effects in household-owned water-holding tanks (the most productive container type and main target for control) was estimated prospectively in two trials. Temephos was applied using spoons or inside perforated small zip-lock bags. Water samples from the study tanks (including positive and negative controls) were collected weekly and subjected to larval mortality bioassays. Water turnover was estimated quantitatively by adding sodium chloride to the study tanks and measuring its dilution 48 hs later.

**Principal Findings:**

The median duration of residual effects of temephos applied using spoons (2.4 weeks) was significantly lower than with zip-lock bags (3.4 weeks), and widely heterogeneous between tanks. Generalized estimating equations models showed that bioassay larval mortality was strongly affected by water type and type of temephos application depending on water type. Water type and water turnover were highly significantly associated. Tanks filled with piped water had high turnover rates and short-lasting residual effects, whereas tanks filled with rain water showed the opposite pattern. On average, larval infestations reappeared nine weeks post-treatment and seven weeks after estimated loss of residuality.

**Conclusions:**

Temephos residuality in the field was much shorter and more variable than expected. The main factor limiting temephos residuality was fast water turnover, caused by householders' practice of refilling tanks overnight to counteract the intermittence of the local water supply. Limited field residuality of temephos accounts in part for the inability of the larval control program to further reduce infestation levels with a treatment cycle period of 3 or 4 months.

## Author Summary

Dengue is currently the most important viral disease of humans transmitted by arthropods worldwide. *Aedes aegypti*, a human-biting mosquito dwelling in artificial domestic containers, is the main vector of dengue. *Ae. aegypti* larval control programs are frequently based on the application of the insecticide temephos. A five-year larval control program in northeastern Argentina significantly reduced infestations but could not maintain them below target levels, especially during summer. Identifying the underlying processes responsible for such shortcomings is important for improving dengue prevention strategies. Large water-holding containers were the most productive container type and the main targets for control. We found that the duration of temephos residual effects in household-owned large tanks was much shorter than expected and allowed early reinfestation post-treatment. The main factor limiting temephos residuality was fast water turnover, caused by householders' practice of refilling tanks overnight to counteract the intermittence of the local water supply.

## Introduction

Dengue is currently the most important arboviral disease in the world; it affects an estimated 50 million people and causes 30,000 deaths per year [1], [2]. In Argentina, the regional context of increasing dengue incidence led in 2009 to the most severe and extended DF epidemic ever recorded [3]. *Aedes aegypti* (Diptera: Culicidae) (L.), the main vector of dengue, urban yellow fever and chikungunya virus, is a highly domestic and anthropophilic mosquito found inside or around human dwellings in urban settings [4]. In the absence of a vaccine, efforts to reduce dengue transmission frequently rely on vector control actions targeting immature stages through chemical or biological treatment of artificial water-holding containers. An international panel recently concluded that strategies for vector control and disease prevention need to be greatly improved [5].

A five-year, city-wide control program for the prevention of dengue transmission applied temephos in granular formulation to water-holding containers using spoons every 3 or 4 months in Clorinda, northeastern Argentina, from 2003 to 2008 [6]. The program successfully limited dengue transmission and significantly reduced larval indices but failed to maintain them below target levels (the city-wide Breteau index was rarely <5%). Large tanks were found to be the most productive type of water-holding container [7], as in several studies around the globe [8]–[11]. Significant larval resistance to temephos was not recorded locally [12]. Incomplete surveillance coverage and reduced residuality of temephos under local conditions were held as the main putative factors limiting the effectiveness of control actions [6].

The duration of the residual effects of a given treatment (i.e. the amount of time the treatment is effective for vector control after its application) is a very important metric needed to estimate the frequency of treatment applications required to achieve control objectives. Under field conditions, treatments are affected by site-specific processes that modify the duration of residual effects relative to what is measured in controlled experiments under more artificial conditions. Therefore, the ultimate evaluation of treatment effectiveness is under field conditions [13].

Temephos, an organophosphate insecticide not toxic for humans at recommended doses, has been extensively used as a larvicide against *Ae. aegypti* during the past 40 years [14]–[18]. It is generally applied in granular formulation and delivered into containers using spoons, and more recently, inside permeable bags for slow release and reintroduction after householders clean treated containers [19]. Reference publications traditionally considered that temephos residual effects lasted between 8 and 12 weeks [20] or about 5 weeks [21]. A recently published guide for dengue vector control indicated: "Two or three application rounds carried out annually in a timely manner with proper monitoring of efficacy may suffice, especially in areas where the main transmission season is short." [22]. Recent studies using different temephos formulations, application procedures and experimental conditions have shown widely variable durations of residual effects ranging from 1 to 6 months [19], [23], [24]. The actual residuality of temephos under field conditions has rarely been documented. Infestation was detected within 7 days post-treatment with temephos in Brazil [25] and Nicaragua [26], but in the former study the number of experimental units was very limited (<18) whereas in the latter containers from 1,903 study houses were treated without the supervision of the investigators and observed only once post-treatment. In Peru, the larvicidal effect of temephos started to decline 7 weeks post-treatment but the field study only included eight experimental units [23]. None of these studies sought to identify the processes that caused such limited, widely variable effectiveness of temephos.

Our field-based study conducted in Clorinda had four objectives: (i) Estimate the duration of the residual effects of temephos in large water-storage tanks by means of larval mortality bioassays; (ii) Compare the effectiveness of temephos applied with spoons or inside permeable zip-lock bags; (iii) Identify factors and processes associated with the eventual decay of temephos residuality, and (iv) Describe the temporal pattern of *Ae. aegypti* immature infestation in containers treated with temephos.

## Materials and Methods

### Study site

A larval control program was run by Fundación Mundo Sano (FMS) and other organizations in Clorinda (lat 25°17′S, long 57°43′W), northern Argentina, from 2003 to 2008 [6]. The city had nearly 50,000 inhabitants in 2008. This study was carried out in Primero de Mayo, a large neighborhood with 2,500 houses (20% of the city) and relatively high infestation levels [7]. This neighborhood has an intermittent piped (tap) water service; ground-level, water-storage tanks (300-1,000 L) made of fibrocement or plastic are found in almost 50% of the lots (mean, 1.3 ground-level tanks per lot). The water harbored by these containers is used for many different purposes (e.g., washing, drinking, bathing, cooking, watering plants).

### Study design

Two longitudinal studies (pilot and main trial) assessed the larvicidal effects of temephos in large water-holding tanks. Mortality bioassays of *Ae. aegypti* larvae exposed to water samples collected from treated and control containers at several occasions post-treatment were performed. Containers were selected randomly from a database that included approximately 1,300 large tanks identified by lot, size and material in 2007 [7].

During each trial, lots with selected containers were visited and the proposed activity was explained to the head of each household who was asked to give oral consent for temephos treatment, following customary practices of the ongoing larval control program since 2003 [6]. If permission was granted, consent was recorded in a form and each tank was treated with temephos at the recommended dose of 1 ppm (1% granular formulation, Fersol) by experienced FMS field personnel who regularly conduct vector control operations in the area. Following treatment, water samples from each study tank were collected weekly into 500 ml glass jars. Prior to collection, the water of each tank was stirred. Each jar was placed in expanded polystyrene thermic boxes and transported to the local FMS laboratory. This procedure is very similar to the one described by Palomino and others [23]. Two control tanks, one positive (treated with temephos) and one negative (untreated), were prepared in 300 liter-fibrocement tanks filled with piped water and then kept fully lidded and protected from rain and direct sunlight at the backyard of the laboratory.

Immediately after the arrival of water samples to the laboratory, mortality bioassays were performed by exposing 20 third- or fourth-instar larvae of *Ae. aegypti* to each water sample and recording mortality 24 hs later. The glass jars were left unlidded during the bioassays. Similar methodologies have been used previously [17], [23]–[27]. The larvae used were the second generation of larvae collected in a randomly selected block of Primero de Mayo in 2007 and were reared at the local FMS laboratory. A temephos-treated container was considered to have lost larvicidal effects when bioassay larval mortality was <70% [17], [24], [27].

### Pilot trial

A pilot trial was conducted in order to test the procedures and estimate how many water samples per container were necessary. A total of 18 selected tanks was treated with temephos applied using spoons on February 4, 2008 (mid-summer). Duplicate water samples were successfully collected weekly during 5 weeks post-treatment; positive controls were tested twice on weeks 7 and 8 post-treatment. Half of the containers were not sampled in the first week post-treatment due to unforeseen operational constraints (i.e., not enough larvae were produced). Meteorological data were collected by a local weather station run by Cooperativa de Provisión de Obras y Servicios Públicos Clorinda Limitada. During this trial, the mean temperature was 26.1°C and mean daily maximum and minimum temperatures were 31.8°C and 23.8°C, respectively. Cumulative rainfall was 151.6 mm.

### Main trial

Sixty water-storage tanks not included in the pilot trial (mean volume, 400 L; standard deviation, 198 L) were treated with temephos applied either using spoons or inside permeable zip-lock bags on November 5, 2008 (mid-spring), and followed up for 14 weeks until February 14, 2009 (mid-summer). Containers were randomly assigned to each treatment in a balanced design. When zip-lock bags were used, holes were punched with a paper clip prior to treatment, and householders were instructed to reintroduce the bag after eventually cleaning or emptying the container. Further discussion on water use practices was not engaged in order to minimize behavioral changes or create any bias toward the study containers.

Water samples for bioassays were collected immediately before treatment, at 2 days post-treatment, and at weekly intervals until reaching 14 weeks post-treatment; samples were not collected at week 8 post-treatment (extending over Christmas). Based on bioassay results from the pilot trial and other tests performed, only one sample of water per container was taken at each time point (data not shown). During the first visit to each study lot, each container was scored for sun exposure (considered low if any structure such as a ceiling or tree overshadowed the container, or high otherwise); container material (fibrocement or plastic), and water type (only rain water, only piped water, or rain and piped water). Pump water was rarely used in the study neighborhood, and not used at all in the randomly selected tanks included in the main trial. Positive and negative control tanks were stirred weekly because preliminary results suggested that the larvicide became attached to the fibrocement of the tanks [28].

The presence or absence of larvae and the number of pupae were registered every time a water sample was collected during the follow-up. All pupae and larvae were collected with large-mouth pipettes; frequently the operators used small sieves in order to strain the container and collect samples of immatures. Samples were placed in labeled test tubes and transported to the laboratory for processing. Larvae were identified to species level using an entomological magnifying glass and an illustrated key [29]. Pupae were kept in small water-filled plastic vials until emergence to allow accurate species identification as adults.

During the first seven weeks of the main trial, the larval control program team visited the rest of the houses in the study neighborhood and either removed, emptied or treated all water-holding containers found with temephos; the study tanks included in the main trial were excluded from these regular operations. The larval control program had not treated the neighborhood during the previous six months. During the main trial, the mean temperature was 25.9°C and mean daily maximum and minimum temperatures were 31.9°C and 20.4°C, respectively. Mean monthly cumulative rainfall was 123.5 mm/month.

To describe (re)infestation patterns, for each of the tanks in the main trial the temporal differences between the following events were calculated: treatment; first occurrence of infestation post-treatment; treatment of the block (by other larval control program teams) where the tank was located, and loss of residual effects.

### Water turnover

The intensity of water turnover in tanks of the main trial was estimated by a procedure based on adding 100 ppm of sodium chloride to the containers and measuring the dilution of chloride during the subsequent 48 hs after concluding the follow-up (at week 16 post-treatment). Sodium chloride was selected because both sodium and chloride ions are natural water components and are not expected to suffer significant chemical transformations within 48 hs. The selected concentration was 100 ppm because preliminary estimates of typical chloride concentration in the study tanks were <50 ppm, and the final concentration would be below half of the taste threshold of sodium chloride (considered to be 300 ppm [30]). Tanks were excluded from water turnover assays if a householder was hypertense and drank water from the tank or if the tank was filled with less than 10% of its capacity. The water turnover assays were performed in 32 of the 60 tanks. Prior to the application of sodium chloride, the procedures and purpose of the study were explained to the head of each household who signed an informed consent. The procedures were approved by the Ethics Committee “Doctor Virgilio G. Foglia” in Buenos Aires, Argentina.

Sodium chloride was added to the tanks from a highly concentrated stock solution. Prior to the addition of the salt, the volume of water held by each container was estimated based on tank diameter and height of the water column. Three samples of water were collected from each tank: the first one at five days before addition of sodium chloride; the second one immediately before addition (both samples were used to estimate the mean basal concentration of chloride), and the third one at 48 hs post-addition of the salt. Water samples were placed in 15 ml-plastic tubes sealed hermetically, stored frozen and transported to Buenos Aires.

Chloride concentration was determined by ionic chromatography in a DIONEX DX-100 equipment with conductivity detector, suppressor, sample injection valve and 25 µL sample loop. Two plastic anion columns were coupled in series to serve both as pre-column and analytical chromatographic column (DIONEX AG-4 and AS-4, respectively). A mixture of HCO_3_^-^/CO_3_^2-^ (1.7 mM/1.8 mM) was chosen as eluent with a flow rate of 2 mL/min. The typical experimental error was lower than 5% for all results.

The volumetric output flow rate (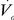) was calculated by considering mass balance equations in each tank [31]. Simplifying flow rates as continuous processes:
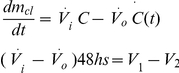



By solving these equations, the volumetric output flow rate may be expressed as:
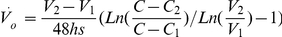
where m_cl_ is the mass of chloride; 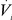 is the volumetric input flow rate; *C* is the concentration of chloride in the water added during the 48 hs post-addition of chloride; *C(t)* is the concentration of chloride over time; *V_1_* is the volume of water measured immediately before the addition of sodium chloride; *V_2_* is the final volume of water 48 hs post-addition of sodium chloride; *C_1_* is the concentration of chloride after adding the salt (calculated as the sum of the concentration observed immediately before addition and 100 ppm −the added concentration), and *C_2_* is the final concentration of chloride 48 hs post-addition of sodium chloride. *C* (unknown) was assumed to equal the mean basal concentration of chloride in each tank over the two pre-application samples. Water turnover intensity was estimated as the volumetric output flow rate multiplied by 48 hs (e.g., the estimated volume of water removed from the tank during the 48 hs) divided by the volume of each tank.

To confirm that these procedures provided valid estimates of water turnover, controlled experiments were performed using small plastic and fibrocement containers filled with running water from Buenos Aires. Five containers were filled with running water and 100 ppm of chloride were added to each of them. A known fraction of the water was replaced with piped water in each container 24 hs later. The resulting concentration of chloride was measured 48 hs post-addition of chloride. The estimated results differed by less than 5% in all cases (Table S1).

The intensity of water turnover was also assessed during the inspection of all houses in the study neighborhood in November-December 2008. At each house visit, the larval control team asked householders on how often they added water to each water-storage tank inspected.

### Data analysis

Ninety-five per cent confidence intervals (CI) for mean larval mortality in the collected water samples were calculated with nonparametric bootstraps using the percentile method implemented with package Boot in R 2.70 [32].

To estimate the association between measured covariates and the larvicidal effect of temephos treatments over time, larval mortality in the bioassays was modeled using generalized estimating equations (GEE) [33]; the individual study tanks were considered as the experimental units. GEE models can serve as an extension of generalized linear models to analyze correlated data [34]. The models were computed with procedure xtgee in STATA 9.0 [35]. The best correlation structure was estimated by computing quasi-likelihoods under the independence model criterion (QIC) [36], a statistic analogous to Akaike's Information Criterion but suitable for GEE models. All QICs were computed following the algorithm of Hardin and Hilbe [34] in STATA. The explanatory variables included in the model were temephos application type, sun exposure, container material, water type, water turnover and the two-way interaction between temephos application type and water type or water turnover. The interactions were selected *a priori* because both types of temephos application were expected to be affected differently by water use practices. Because water type and water turnover were significantly associated, multicollinearity problems were detected when all the covariates were included in the model. Since water turnover had nearly 50% fewer observations than water type, only the latter was included in the model initially. Subsequently, we replaced water type with water turnover to check whether the outcome was robust to the exact specification of water management practices. An association with reduced larval mortality indicates shorter residual effects of temephos. The duration of residual effects for each tank in the main trial was calculated as the number of days until the loss of larvicidal effects (i.e., bioassay larval mortality <70%) was detected.

In the water turnover assays, the estimated final concentration of chloride 48 hs post-addition of sodium chloride (*C_2_*) was lower than the mean basal concentration of chloride in 25% of the cases. In such cases *C* was assumed to equal the minimum value of the two pre-application samples, because the basal concentration of chloride in piped water was highly variable over time and variations in the concentration of chloride during the potabilization process are typically substantial.

## Results

In the pilot trial, the duration of the residual effects of temephos was much shorter than expected and very heterogeneous between individual field tanks. The water collected from one of the tanks did not kill any larvae at the first week post-treatment. Mean larval mortality successively declined to 78% (CI, 46–100%) at one week post-treatment to 83% (CI, 66–100%) at week two, 68% (CI, 50–85%) at week three, and 50–52% (CI, 31–72%) at four or five weeks post-treatment. Only 1% of the larvae from the negative control tank died, whereas the positive control tank showed complete loss of larviciding power at four and five weeks post-treatment. A week later, its water was stirred and subsequent bioassays evidenced full larviciding power. Larval mortality between duplicate water samples was highly correlated (r = 0.97, n = 81, P<0.001).

In the main trial, water samples were successfully collected on 94% of the occasions (n = 849); 4% of the times the container was found dry, and in 2% the owner either refused access or was absent at the time of visit. The positive control tank had 99% larval mortality and the negative control only 1% mortality during the entire follow-up. Larval mortality in water samples collected prior to treatment was nil or close to zero in every container.

**Figure 1:**
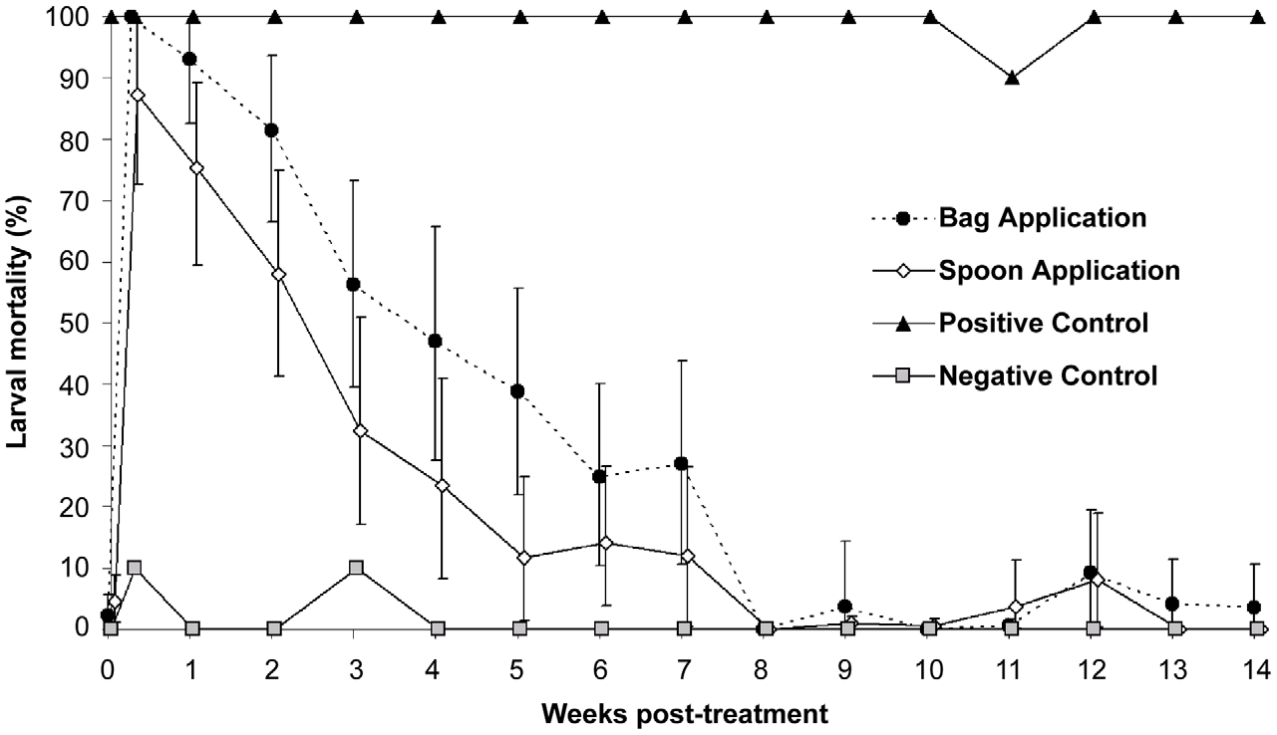
Larval mortality in the bioassays. Mean larval mortality over time after bag- or spoon-based application of temephos in the main trial, Clorinda 2008/2009. Error bars indicate 95% bootstrap confidence intervals. Bag- and spoon-based application curves have been displaced over the x axis to improve visualization.

The residual effects of temephos in spoon-treated containers were shorter than in the pilot trial and also very heterogeneous between individual tanks (Fig. 1). Two of the 30 treated containers showed no larvicidal effects at two days post-treatment. Mean larval mortality was only 25% at four weeks post-treatment. The duration of residual effects of temephos applied with spoons had a median of 2.4 weeks (first quartile  =  2.0 weeks, third quartile  =  4.1 weeks; range 0.3 to 9 weeks).

Temephos applied inside zip-lock bags had longer-lasting residual effects than when applied with spoons (Fig. 1). The first evidence of a container losing all larviciding power occurred at two weeks post-treatment. Mean larval mortality was 50% at four weeks post-treatment. Mean larval mortality was consistently higher relative to the spoon-based application until week 8 post-treatment, when almost all containers showed null larvicidal power. The duration of residual effects of temephos applied with zip-lock bags had a median of 3.4 weeks (first quartile  =  2.3 weeks, third quartile  =  7.7 weeks; range 1 to 10 weeks).

**Figure 2:**
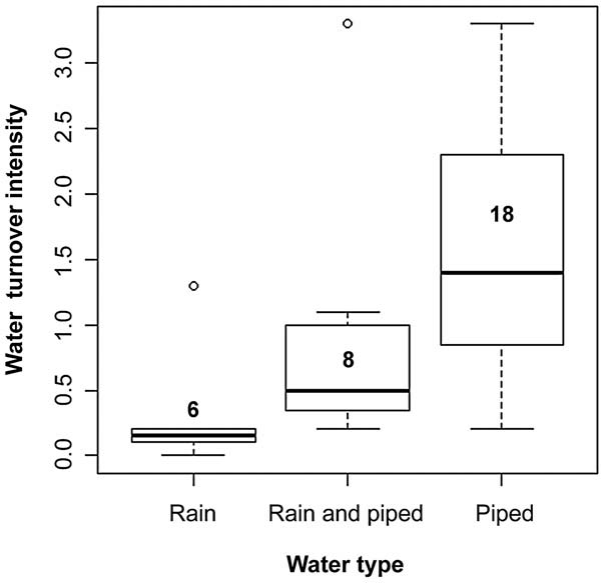
Box-and-whiskers plot of estimated water turnover according to water type. Water turnover was calculated as the estimated volume of water removed during the subsequent 48 hs post-addition of sodium chloride divided by the volume of each tank. The numbers indicate the number of tanks within each category. Main trial, Clorinda, 2009.

Estimated water turnover and water type were highly significantly associated (linear regression, n = 32, F = 8.73, P = 0.001). Containers scored as holding piped water had a very fast, widely variable water turnover; 73% of these containers had an estimated turnover in 48 hs equal to or greater than the container's volume (Fig. 2). In contrast, 85% of tanks containing rain water had an estimated turnover close to 0; an outlier value with high turnover was probably a misclassified container. Containers with rain and piped water showed intermediate turnover rates. No rain occurred during the 48 hs after application of sodium chloride. In the house-to-house survey conducted in late 2008, householders responded that water was added every day in 61% of tanks filled with piped water (n = 582), and every 3 days or less in 86% of them. Among tanks filled with rain water (n = 170), in 94% of the cases householders responded that they waited until it rained for refilling the tanks.

The association between bioassay larval mortality and selected covariates was assessed with multivariate GEE models. The best correlation structure found was a second-order autoregressive correlation. Samples taken from containers with piped water had significantly lower larval mortality than those with other water types (OR = 0.60, CI = 0.48–0.75) (Table 1). Type of temephos application (spoon or zip-lock bag) significantly modified larval mortality depending on water type; reduced larval mortality occurred in spoon-treated containers filled with piped water (OR = 0.43, CI = 0.31–0.60) or with rain and piped water (OR = 0.39, CI = 0.26–0.59) relative to spoon-treated containers filled with rain water. Larval mortality was marginally significantly associated with sun exposure (OR = 1.17, CI = 1.01–1.36). Container material did not exert significant effects on larval mortality when other factors were taken into account. When water turnover was included in the model instead of water type (which reduced sample size by nearly 50%), the results obtained were qualitatively similar except for sun exposure which had insignificant effects (Table S2).

**Table 1:** Odds ratios of the explanatory variables used in the multivariate GEE model of bioassay larval mortality.

Explanatory variables	Odds Ratio	95% confidence interval		*P*-value
Temephos application type				
Bag	1			
Spoon	1.23	0.95	1.60	0.12
Water type				
Rain	1			
Rain and piped	0.91	0.69	1.21	0.53
Piped	0.60	0.48	0.75	0.00
Type of temephos application X Water type				
Spoon X Rain	1			
Spoon X Rain and piped	0.39	0.26	0.59	0.00
Spoon X Piped	0.43	0.31	0.60	0.00
Sun exposure				
High	1			
Low	1.17	1.01	1.36	0.04
Container material				
Fibrocement	1			
Plastic	0.97	0.82	1.15	0.76

Main trial, Clorinda 2008–2009.

The duration of residual effects was used as a summary value of treatment effectiveness according to water type and temephos application type (Table 2). In containers filled with rain water, median residuality was substantially higher than in containers with other types of water. Bag-based applications had a higher median residuality than spoon-based applications in containers filled with rain and piped water.

**Table 2:** Duration of residual effects of temephos according to water type and temephos application type.

		Duration of residual effects (weeks)				
Water type	Type of application	Median	Minimum	First quartile	Third quartile	Maximum
Rain						
	Spoon	6.0	5.0	6.0	9.0	9.0
	Bag	6.0	2.0	4.9	8.5	9.0
	Total	5.9	2.0	4.9	9.0	9.0
Rain and piped						
	Spoon	2.0	2.0	2.0	2.8	4.0
	Bag	6.0	3.0	3.0	9.0	10.0
	Total	3.0	2.0	2.0	4.9	10.0
Piped						
	Spoon	2.0	0.3	0.9	3.9	5.0
	Bag	3.0	1.0	2.0	3.6	9.0
	Total	2.0	0.3	2.0	3.9	9.0

Main trial, Clorinda 2008–2009.

**Figure 3:**
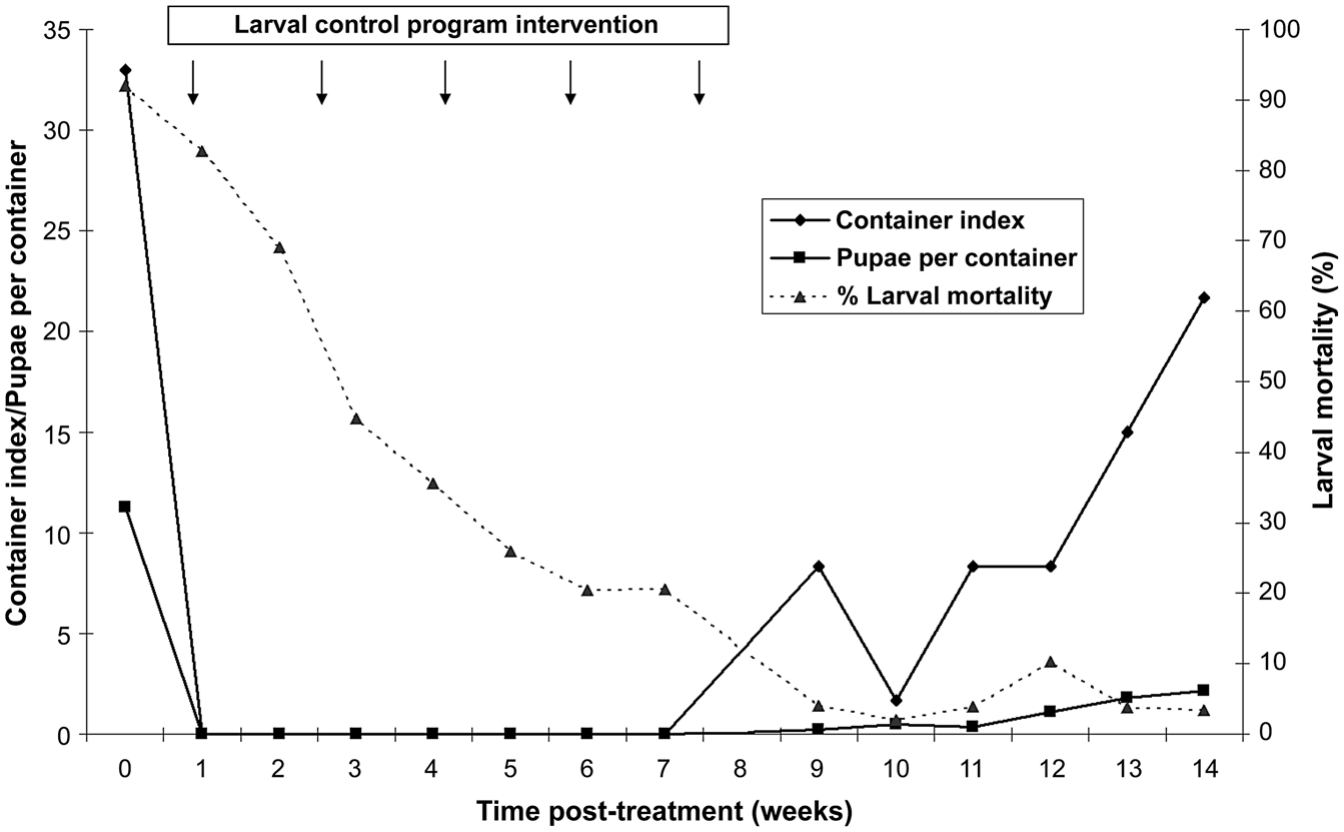
Infestation during the follow-up. Container index, mean number of pupae per container and mean bioassay larval mortality in the main trial over time after bag- or spoon-based application of temephos. Clorinda, 2008/2009.

In the study containers, infestation before treatment was high (container index  =  33% and >10 pupae per container) and dropped to zero immediately after treatment (Fig. 3). The first signs of infestation post-treatment were detected at week 9 (i.e., only two weeks after the larval control program finished treating the entire neighborhood), when bioassay larval mortality dropped below 20%. Infestation continued to rise until the end of the follow-up. There was a significant association between infestation of individual containers before and after treatment with temephos (χ^2^ test, d.f. = 1, P<0.001). Among containers ever found to be infested after treatment, 88% had been infested before treatment. Among containers that were infested just before treatment, 73% became reinfested later. Only 9% of the containers not infested before treatment became infested after treatment. Containers were found to be newly infested at an average of 7 weeks (range 2–12 weeks) after estimated loss of larvicidal effects. However, in containers filled with rain water new infestations appeared on average 3 weeks after loss of larvicidal effects, whereas in containers filled with rain and piped water or only piped water the average time elapsed was much longer (7 and 11 weeks, respectively).

## Discussion

Both trials independently showed that the median duration of temephos residual effects in large water-holding tanks under field conditions (2–3 weeks in the main trial) was much shorter than the expected 5 to 8–12 weeks [20], [21], and widely variable between tanks regardless of type of temephos application. This is consistent with early (re)infestations in Clorinda being recorded 5–7 weeks after treatment with temephos during 2003–2005 [6] and 3 weeks post-treatment in 2008 (Garelli et al., unpublished data). These are important results for larval control programs, especially because temephos is widely used and the very few published reports of residual effects under field conditions show conflicting outcomes for undefined reasons. Secondly, the duration of residuality was modified substantially by water management practices (represented by water type and water turnover) —a novel finding in the dengue mosquito control literature. This short, widely variable residuality of temephos coupled with incomplete surveillance coverage [6] are probably the main causes of the inability of the larval control program to bring infestation below target levels with a treatment cycle period of 3–4 months.

Water type and water turnover were strongly and significantly associated. Multivariate analyses showed that both variables were associated with reduced larval mortality and shorter residuality of temephos. Containers filled with piped water had high water turnover rates and significantly shorter residual effects, especially when temephos was applied with spoons. Conversely, containers filled with rain water had lower turnover and much longer residual effects despite the occurrence of rainfall during each of the first six weeks of the main trial. Tanks filled with rain and piped water had intermediate water turnover rates, and residuality significantly differed between bag- and spoon-based applications. The interaction between water type and temephos application type was expected because zip-lock bags with undissolved insecticide were probably reintroduced after containers were emptied (bags were observed inside the tanks throughout the follow-up) [19] and thus provided extended residuality. Similar associations are expected to occur regardless of the larvicide used because high water turnover implies increased clearance of larvicides and shorter residual effects.

The intense water turnover rate in tanks filled with piped water may be explained by current water management practices determined by the intermittent local water supply, especially during the hot summer months when water consumption increases. In the study area, piped water was generally available during the night and was very limited or unavailable during daytime. Many householders reported refilling their tanks every night or so and, in some cases, emptying the tanks before refilling them. Conversely, containers filled with rain water had a much more irregular filling regime dependent on rainfall and almost no water turnover. These observations explain the strong association between water type and water turnover. Based on direct and indirect estimates of water use (Text S1) and householders' reports, we infer that local water management practices determined fast water turnover rates and caused the short-lasting residual effects of temephos in both field trials.

The effects of water management practices on *Ae. aegypti* abundance under recurrent larval control actions are complex and probably nonlinear. Intensely managed containers with fast water turnover (e.g., as those filled with piped water) are expected to have short-lasting chemical residual effects, but its suitability for adult *Ae. aegypti* production also depends on the process by which the tank is refilled. If the container is emptied often then it may not become suitable for adult mosquito production because immature stages are eliminated before adult emergence, but if water is added without removing or overflowing immature stages from the container it may become a suitable breeding site. Containers with low or very sporadic water turnover, such as those filled with rain water, should (and did) retain temephos residual effects for much longer periods. However, in the absence of effective surveillance and treatment or after residual effects wane, containers filled with rain water would become the most productive type if other conditions for suitability hold. Rainwater-filled containers became reinfested faster (mean, 3 weeks) than containers with at least piped water (mean, 7–11 weeks), and in a previous survey they had greater probability of being infested and produced more pupae [7].

The most likely sources of reinfestation post-treatment were breeding sites left untreated in closed premises or where householders refused interventions [6]. Even though the existence of putative cryptic sites cannot be completely excluded [37], intensified searches for them yielded negative results [6]. Studies with molecular markers are needed to provide concluding evidence on whether the detected (re)infestations post-interventions were persistent residual foci from eggs surviving treatment or new infestations from genetically different mosquitoes.

Containers infested before treatment were the most likely (73%) to become infested post-treatment, whereas a small fraction of those not infested before treatment (9%) became newly infested. This pattern suggests that the determinants of container suitability for mosquito breeding remained mostly invariant after interventions. In a previous study, containers located in yards rather than indoors, at low sun ex posure, unlidded, filled with rain water, and holding polluted water were found to be positively associated with infestation by larvae or pupae [7]. Most of these factors are related to householders' practices and may likely remain stable over time and space.

Our study has several limitations. Traditional bioassays measure larval survival after 24 hr whereas inhibition of adult production would be the epidemiologically significant metric for assessing transmission risk. If the larvae that survived treatment failed to develop into adults, the actual duration of temephos residual effects with respect to adult production would be underestimated. A larger number of study tanks would have allowed increased precision of larval mortality and associated parameter estimates. Although conclusions drawn from water turnover estimates would have been better supported with a larger sample size and more replicates for each container, the outcome was consistent with householders' reports on how often they managed their tanks and other indirect estimates. Water turnover estimates were probably imprecise because basal concentrations of chloride were widely variable between and within tanks. However, this relative imprecision would not compromise our main conclusions given the large differences in temephos residuality according to water type or water turnover rate. Post-treatment infestation in the main trial was probably underestimated because weekly operations removed all pupae and a sample of larvae, and perhaps very frequent house visits may have promoted householders' awareness and elimination of immature stages from the containers. These processes may also explain the lower number of pupae per container after treatment relative to pre-treatment levels. Had oviposition in the study tanks been monitored, it would have provided valuable information on the links between mosquito vital parameters and temephos treatment.

The positive control tank during the pilot trial had a transient loss of larviciding effects that were recovered after stirring its water. The tendency of temephos to attach to container walls was regarded as a positive feature derived from slow-release properties [38]. In practice, completely unmanaged tanks (i.e. without water movement) were very rare and therefore attachment of temephos to walls may only occur marginally. In the main trial, the water in the positive control tank was periodically stirred and achieved 99% larval mortality through the 14-week period, thus proving the efficacy of the larvicide and the absence of temephos resistance in Clorinda [12].

### Implications for *Ae. aegypti* larval control

The wide gap between expected and actual durations of temephos residual effects under field conditions relative to average treatment cycle periods (3–4 months) accounts in part for not meeting larval control targets in Clorinda and probably elsewhere in northern Argentina.

Our results underline the importance of considering water use practices for the case of dengue, and local specificities in general, when designing, testing and implementing control interventions. Most results in epidemiology are context-dependent [39]. Unconditional recommendations may be misleading and undermine the effectiveness of larval control programs. More importantly, our present results and the outcome of the five-year larval control program [6] cast serious doubt on whether two or three application rounds of larvicides carried out annually in a timely manner [22] would be sufficient to achieve control target levels in many settings such as ours.

Water use practices depend on cultural patterns and water availability, and all three constitute a complex set of factors affecting dengue transmission dynamics [40], [41]. Environmental modifications such as the installation of a reliable piped water service are one of the principal actions for dengue vector control [22], [42]. Further research is needed to better understand the links between water management practices, dengue vector control, mosquito abundance and viral transmission. Temephos application inside small zip-lock bags extended the duration of residual effects relative to spoon-based applications; it was well received by householders, and was easily and inexpensively implemented. However, it is insufficient to achieve larval control goals with a treatment cycle period of 3 or 4 months. Considering the observed rate of reinfestation, a treatment cycle of 2 months would greatly improve larval control status at the expense of almost doubling labor costs and increasing community fatigue. The feasibility and sustainability of such high-frequency cycle periods in cities the size of Clorinda remain questionable. Novel forms of applying the larvicide specifically designed to cope with fast water turnover or new slow-release agents and formulations are needed to improve current larval control tactics. Biological control agents such as fish or cyclopoid copepods [43] may also be appropriate for this type of context. A different approach derived from present findings would seek to incorporate water use practices as control measures. Depending on how the intense management of tanks is performed, a strategy based on community participation aiming at healthier household water management practices may reduce infestations substantially because most water-storage tanks had piped water (77%, 582/752) and therefore were subjected to intense water management. Integrated control interventions capturing the multifaceted nature of *Ae. aegypti* population dynamics have the potential to achieve a much improved vector control status and prevention of dengue transmission.

## Supporting Information

Text S1 Comparison between estimated water turnover intensity in this study and an indirect estimation based on mean water consumption in Clorinda.
(0.02 MB DOC)

Table S1 Basal concentration of chloride (C), volume of solution after adding the salt (V1), concentration of chloride after adding the salt (C1), actual volume of solution removed, actual volume of water added, final volume of solution 48 hs post-addition of sodium chloride (V2), final concentration of chloride 48 hs post-addition of sodium chloride (C2), estimated volumetric output flow rate (V0) and estimated volume of water removed in 48 hs (V0*48 hs) in containers used in the controlled experiments performed to validate water turnover methods.
(0.02 MB DOC)

Table S2 Odds ratios for the explanatory variables used in the multivariate GEE model of bioassay larval mortality that included water turnover instead of water type. Main trial, Clorinda 2008–2009.
(0.03 MB DOC)

Alternative Language Abstract S1 Translation of the abstract into Spanish by author FMG.
(0.02 MB DOC)
