# Supplementary material for: Water Use Practices Limit the Effectiveness of a Temephos-Based Aedes aegypti Larval Control Program in Northern Argentina
Source: PLoS Negl Trop Dis. 2011 Mar 22;5(3):e991. doi: 10.1371/journal.pntd.0000991 (PMC3062537; doi:10.1371/journal.pntd.0000991)
Supplement: Text S1 — Comparison between estimated water turnover intensity in this study and an indirect estimation based on mean water consumption in Clorinda. (0.02 MB DOC) [file pntd.0000991.s001.doc]

**Text S1**

The intense water turnover in the study tanks is roughly consistent with an indirect estimate based on the mean daily consumption of water in Clorinda during summer (250-300 L per capita, according to the local water supply service); mean household size in the study neighborhood (4 people); mean volume of water-storage tanks (400 L), and mean number of tanks per household (1.3). To satisfy most of the demands of an average household during summer, the water-storage tanks would have to be filled up approximately twice a day if enough tap water was provided and no other sources of water were used.
