# Supplementary material for: Water Use Practices Limit the Effectiveness of a Temephos-Based Aedes aegypti Larval Control Program in Northern Argentina
Source: PLoS Negl Trop Dis. 2011 Mar 22;5(3):e991. doi: 10.1371/journal.pntd.0000991 (PMC3062537; doi:10.1371/journal.pntd.0000991)
Supplement: Table S1 — Basal concentration of chloride (C), volume of solution after adding the salt (V1), concentration of chloride after adding the salt (C1), actual volume of solution removed, actual volume of water added, final volume of solution 48 hs post-addition of sodium chloride (V2), final concentration of chloride 48 hs post-addition of sodium chloride (C2), estimated volumetric output flow rate (V0) and estimated volume of water removed in 48 hs (V0*48 hs) in containers used in the controlled experiments performed to validate water turnover methods. (0.02 MB DOC) [file pntd.0000991.s002.doc]

**Table S1. Basal concentration of chloride (*C*), volume of solution after adding the salt (*V_1_*), concentration of chloride after adding the salt (*C_1_*), actual volume of solution removed, actual volume of water added, final volume of solution 48 hs post-addition of sodium chloride (*V_2_*), final concentration of chloride 48 hs post-addition of sodium chloride (*C_2_*), estimated volumetric output flow rate (*V_0_*) and estimated volume of water removed in 48 hs (*V_0_**48 hs) in containers used in the controlled experiments performed to validate water turnover methods.**

| Container | *C* | *V_1_* | *C_1_* | Volume substracted | Volume added | *V_2_* | *C_2_* | $\mathop{V}\limits^{.} _{o}$ | $\mathop{V}\limits^{.} _{o}$*48 hs |
| --- | --- | --- | --- | --- | --- | --- | --- | --- | --- |
| Plastic 1 | 47 | 0.9 | 147 | 0.50 | 0.70 | 1.1 | 97 | 0.01 | 0.49 |
| Plastic 2 | 47 | 0.8 | 147 | 0.66 | 0.86 | 1.2 | 82 | 0.01 | 0.64 |
| Fibrocement 1 | 47 | 1.9 | 147 | 1.00 | 1.20 | 2.1 | 102 | 0.02 | 0.99 |
| Fibrocement 2 | 47 | 1.9 | 147 | 1.04 | 1.24 | 2.1 | 101 | 0.02 | 1.03 |
| Fibrocement 3 | 47 | 1.8 | 147 | 1.24 | 1.44 | 2.2 | 90 | 0.03 | 1.28 |
