# Supplementary material for: Water Use Practices Limit the Effectiveness of a Temephos-Based Aedes aegypti Larval Control Program in Northern Argentina
Source: PLoS Negl Trop Dis. 2011 Mar 22;5(3):e991. doi: 10.1371/journal.pntd.0000991 (PMC3062537; doi:10.1371/journal.pntd.0000991)
Supplement: Table S2 — Odds ratios for the explanatory variables used in the multivariate GEE model of bioassay larval mortality that included water turnover instead of water type. Main trial, Clorinda 2008–2009. (0.03 MB DOC) [file pntd.0000991.s003.doc]

**Table S2. Odds ratios for the explanatory variables used in the multivariate GEE model of bioassay larval mortality that included water turnover instead of water type. Main trial, Clorinda 2008-2009.**

| Explanatory variables | | Odds Ratio | 95% confidence interval | | *P*-value |
| --- | --- | --- | --- | --- | --- |
| Temephos application type | | |  |  |  |
|  | Bag | 1 |  |  |  |
|  | Spoon | 1.24 | 0.85 | 1.27 | 0.12 |
| Water turnover | | 0.77 | 0.67 | 0.90 | 0.001 |
| Type of temephos application X Water turnover | | | |  |  |
|  | Bag X Water turnover | 1 |  |  |  |
|  | Spoon X Water turnover | 0.72 | 0.57 | 0.91 | 0.006 |
| Sun exposure | |  |  |  |  |
|  | High | 1 |  |  |  |
|  | Low | 1.04 | 0.85 | 1.27 | 0.69 |
| Container material | | |  |  |  |
|  | Fibrocement | 1 |  |  |  |
|  | Plastic | 1.14 | 0.92 | 1.42 | 0.22 |
