# Supplementary material for: Water Use Practices Limit the Effectiveness of a Temephos-Based Aedes aegypti Larval Control Program in Northern Argentina
Source: PLoS Negl Trop Dis. 2011 Mar 22;5(3):e991. doi: 10.1371/journal.pntd.0000991 (PMC3062537; doi:10.1371/journal.pntd.0000991)
Supplement: Alternative Language Abstract S1 — Translation of the abstract into Spanish by author FMG. (0.02 MB DOC) [file pntd.0000991.s004.doc]

***Antecedentes:*** En Clorinda, una ciudad del norte de Argentina, un programa de control basado en la aplicación regular del larvicida temephos en toda la ciudad durante 5 años redujo significativamente los índices larvales de *Aedes aegypti* pero no logró mantenerlos por debajo de los valores recomendados. Para explicar la efectividad limitada de las acciones de control, se postuló como posibles causas a la incompleta cobertura y una reducción en la residualidad del temephos.

***Metodología:*** La duración del efecto residual del temephos en tanques grandes (el tipo de recipiente más productivo y el principal objetivo de control) se estimó en forma prospectiva en dos ensayos. El temephos se aplicó utilizando cucharas o dentro de pequeñas bolsas “zip-lock” perforadas. Se colectaron semanalmente 1muestras de agua de los tanques en estudio (incluyendo controles positivos y negativos) y con esas muestras se realizaron bioensayos de mortalidad de larvas. El recambio de agua en los tanques en estudio se estimó cuantitativamente mediante la adición de cloruro de sodio y la medición de su dilución 48 hs después.

***Resultados:*** La duración mediana del efecto residual del temephos aplicado con cucharas (2,4 semanas) fue significativamente menor que aplicado dentro de las bolsas “zip-lock” (3,4 semanas), y fue muy heterogénea entre los tanques. Modelos del tipo “generalized estimating equations” mostraron que la mortalidad de larvas en los bioensayos se vio fuertemente afectada por el tipo de agua y la forma de aplicación del temephos dependiendo del tipo de agua. El recambio y el tipo de agua estuvieron fuerte y significativamente asociados. Los tanques llenados con agua de red mostraron altas tasas de recambio de agua y corta duración del efecto residual, mientras que los tanques llenados con agua de lluvia mostraron el patrón opuesto. En promedio, la infestación larval reapareció nueve semanas post-tratamiento y siete semanas luego de que se detectara la pérdida del efecto residual.

***Conclusiones:*** La residualidad del temephos en condiciones de campo fue mucho menor y más variable que lo esperado. El principal factor limitante de la residualidad fue el rápido recambio de agua, que ocurre dado que los habitantes del barrio rellenan sus tanques durante la madrugada para compensar la intermitencia del servicio de agua de red. La duración limitada de la residualidad del temephos en condiciones de campo da cuenta en parte de la incapacidad del programa de control para reducir en mayor medida los niveles de infestación mediante un ciclo de tratamientos de tres o cuatro semanas.
